# Male Infertility: A Comprehensive Review of Urological Causes and Contemporary Management

**DOI:** 10.3390/jcm15010397

**Published:** 2026-01-05

**Authors:** Biagio Barone, Ugo Amicuzi, Simone Tammaro, Michelangelo Olivetta, Marco Stizzo, Michele Musone, Luigi Napolitano, Luigi De Luca, Pasquale Reccia, Federico Capone, Arturo Lecce, Giovanni Pagano, Silvestro Imperatore, Stefano Chianese, Salvatore Papi, Giampiero Della Rosa, Fabrizio Dinacci, Mariano Coppola, Antonio Madonna, Marco Grillo, Dante Di Domenico, Francesco Del Giudice, Vincenzo Francesco Caputo, Dario Del Biondo, Roberto Falabella, Felice Crocetto

**Affiliations:** 1 Department of Urology, P.O. San Paolo, ASL Napoli 1 Centro, 80145 Naples, Italy; grillomarco@inwind.it (M.G.); dantedid@gmail.com (D.D.D.); dario.delbiondo@aslnapoli1centro.it (D.D.B.); 2 Department of Neurosciences, Sciences of Reproduction and Odontostomatology, University of Naples Federico II, 80131 Naples, Italy; u.amicuzi@gmail.com (U.A.); simone.tammaro95@gmail.com (S.T.); drmichelemusone@gmail.com (M.M.); nluigi89@libero.it (L.N.); fedecapone@outlook.it (F.C.); arturo.lecce92@gmail.com (A.L.); giovanni.pagano1@outlook.it (G.P.); silvestro.imperatore97@gmail.com (S.I.); stechianese99@gmail.com (S.C.); salvatorepapi@blu.it (S.P.); giampierodellarosa@gmail.com (G.D.R.); fabrizio.dinacci18@gmail.com (F.D.); marianocoppola99@icloud.com (M.C.); antoniomadonna1997@icloud.com (A.M.); felice.crocetto@unina.it (F.C.); 3 Urology Unit, Department of Surgical Sciences, AORN Sant’Anna e San Sebastiano, 81100 Caserta, Italy; olivetta.drmichelangelo@gmail.com; 4 Urology Unit, Department of Woman, Child and General and Specialized Surgery, University of Campania Luigi Vanvitelli, 80131 Naples, Italy; marcostizzo@hotmail.com; 5 Division of Urology, Department of Surgical Multispecialty, AORN Antonio Cardarelli, 80131 Naples, Italy; luigideluca86@gmail.com; 6 Urology Unit, AORN Ospedali dei Colli, Monaldi Hospital, 80131 Naples, Italy; reccia.pasquale1@gmail.com; 7 Department of Urology, University Sapienza, 00185 Rome, Italy; francesco.delgiudice@uniroma1.it; 8 Unit of Urology, San Carlo Hospital, 85100 Potenza, Italy; vincitor@me.com (V.F.C.); rfalabella@libero.it (R.F.)

**Keywords:** male infertility, varicocele, erectile dysfunction, Peyronie’s disease, azoospermia, sperm retrieval, lifestyle modifications, urological causes

## Abstract

Male infertility is a prevalent global health issue, with urological disorders representing some of the most common and correctable causes. Key conditions such as varicocele, obstructive azoospermia, erectile dysfunction and Peyronie’s disease impair fertility through distinct pathophysiological mechanisms, including disrupted spermatogenesis, reproductive tract obstruction and failed sperm delivery. The effective management of these conditions hinges on a systematic diagnostic evaluation, which integrates clinical history, physical examination, semen analysis and specialized imaging. Modern management follows a logical progression, beginning with foundational lifestyle modifications, advancing to targeted medical or surgical interventions, and culminating, when necessary, in assisted reproductive technologies. Treatment strategies are therefore highly targeted, ranging from medical management and surgical correction—such as varicocelectomy or microsurgical reconstruction—to sperm retrieval techniques. Furthermore, evidence-based lifestyle modifications and a multidisciplinary clinical approach are fundamental to optimizing reproductive outcomes for affected couples. A comprehensive understanding of these urological etiologies is therefore essential for guiding appropriate intervention and improving the prospects of achieving pregnancy.

## 1. Introduction

Infertility, clinically defined as the failure to achieve a pregnancy after 12 months or more of regular, unprotected sexual intercourse, representing a significant global health challenge [1]. It is estimated to affect approximately 15% of couples worldwide, with a male factor identified as either a primary or contributing cause in nearly half of all cases [2]. This statistic underscores a critical paradigm shift in reproductive medicine. The focus has moved away from solely the female partner toward a more equitable and comprehensive approach. Male reproductive health is now recognized as a pivotal component of the fertility equation. Male infertility is inherently multifactorial, with its origins often lying in a complex interplay of genetic predispositions, hormonal imbalances, environmental exposures and lifestyle choices [3,4]. Within this intricate web of causality, urological disorders emerge as some of the most significant and, importantly, treatable contributors, interfering with the fundamental processes of sperm production, maturation or delivery, making their understanding essential for any clinician in the field [5]. This analysis will concentrate on several key urological conditions that directly impair fertility by disrupting spermatogenesis, sperm transport or ejaculatory function. These include varicocele, one of the most common correctable cause; obstructive azoospermia, where a physical blockage prevents the ejaculation of normally produced sperm; erectile dysfunction (ED), which presents a direct mechanical barrier to conception; Peyronie’s disease (PD), which can physically preclude successful intercourse [6,7,8]. For each condition, a nuanced understanding of the underlying pathophysiology is essential, as it directly informs the diagnostic pathway and guides the selection of appropriate interventions [9]. The management of these conditions showcases the dynamism of modern urology, offering a range of solutions from medical management and lifestyle modifications to microsurgical reconstruction and sperm retrieval techniques for advanced assisted reproductive technologies (ART) [10,11]. The present work aims to provide a comprehensive examination of the epidemiology, pathophysiology, diagnostic workup and contemporary management of these key urological causes of male infertility. By synthesizing the latest evidence and clinical guidelines, this resource is designed to serve clinicians across specialties, empowering them to deliver holistic, effective and timely care that significantly improves the prospects for couples striving to achieve pregnancy.

## 2. Etiological Risk Factors in Male Infertility—Genetics and Environment

The declining trend in male fertility, as evidenced by global reductions in sperm count and quality, is a complex multifactorial issue driven by the dynamic and often deleterious interplay between an individual’s genetic predisposition and a lifetime of acquired exposures [12,13]. The pathogenesis of male infertility is rarely attributable to a single cause but is instead the product of a cumulative burden where genetic susceptibilities can be powerfully exacerbated by environmental, lifestyle and medical insults [14]. A comprehensive understanding of this intricate risk factor landscape is paramount for advancing clinical evaluation, guiding personalized therapeutic strategies and informing public health initiatives aimed at mitigation [15]. Genetic factors represent the fundamental, often immutable, blueprint upon which all reproductive potential is built. These inherent risks can be broadly categorized into chromosomal, monogenic and epigenetic anomalies. Chromosomal abnormalities, such as Klinefelter syndrome (47,XXY), stand as a leading genetic cause of primary testicular failure [16]. The supernumerary X chromosome leads to the progressive hyalinization and fibrosis of the seminiferous tubules, typically resulting in azoospermia and hormonal profiles marked by elevated gonadotropins and low testosterone [17]. Similarly, structural Y-chromosome microdeletions, specifically within the critical azoospermia factor (AZF) regions—AZFa, AZFb and AZFc—directly excise genes essential for various stages of spermatogenesis, with the specific deletion location predicting the severity of the spermatogenic defect [18]. Beyond karyotypic anomalies, specific single-gene disorders have profound implications. Mutations in the cystic fibrosis transmembrane conductance regulator (CFTR) gene are classically associated with congenital bilateral absence of the vas deferens (CBAVD), causing obstructive azoospermia in an otherwise hormonally normal male [19,20]. A growing list of other genes, including those involved in hypothalamic function (e.g., GNRHR, KAL1), sperm structure (e.g., SMPC, AKAP) and DNA repair, are continually being implicated, revealing the intricate genetic architecture that underpins male fertility [21,22,23,24,25]. Furthermore, the emerging field of epigenetics highlights how paternal lifestyle and environmental exposures can lead to transmissible alterations in sperm DNA methylation and histone modification, potentially affecting embryonic development and offspring health [26]. Compounding this genetic susceptibility is a wide array of acquired risk factors that can insidiously or acutely compromise reproductive function. Environmental and occupational exposures act as pervasive threats to spermatogenesis. Endocrine-disrupting chemicals (EDCs), such as pesticides (e.g., dichlorodiphenyltrichloroethane), plastics components (e.g., phthalates, bisphenol A) and industrial pollutants (e.g., dioxins), can interfere with the hypothalamic-pituitary-gonadal axis by mimicking or blocking endogenous hormonal action [27,28]. Physical hazards, including chronic exposure to excessive heat in certain occupations or from sedentary habits and ionizing radiation from medical or industrial sources, directly damage the mitotically active spermatogonia, leading to prolonged or permanent deficits in sperm production [29,30]. Lifestyle choices further modulate this risk profile, creating a modifiable frontier for intervention. Obesity, a pandemic health issue, contributes to infertility through multiple mechanisms, including the peripheral aromatization of androgens to estrogens in adipose tissue, which suppresses gonadotropins release and through the associated systemic inflammation and oxidative stress that damages sperm membranes and DNA [31]. The detrimental effects of smoking are dose-dependent and well-documented; polycyclic aromatic hydrocarbons and other toxins in tobacco smoke not only reduce sperm count and motility but are also strongly linked to increased sperm DNA fragmentation [32]. Similarly, excessive alcohol consumption can suppress testosterone synthesis and lead to testicular atrophy, while recreational drugs like anabolic steroids and cannabis further disrupt the delicate endocrine balance required for fertility [33,34,35]. Finally, the role of advancing paternal age is increasingly recognized; it is associated with a gradual decline in semen parameters, an increase in sperm DNA fragmentation due to diminished DNA repair mechanisms and a heightened risk of de novo genetic mutations in offspring, linking paternal age to a higher incidence of certain neurodevelopmental disorders (Table 1) [36].

## 3. Classification of Male Infertility

Male infertility is a profoundly multifactorial condition, representing a complex and often intertwined web of genetic, physiological, environmental and lifestyle determinants that disrupt the intricate process of sperm production, maturation or delivery. To systematically approach this diagnostic challenge, clinicians rely on a pathophysiological classification system that categorizes cases into three primary domains: obstructive, non-obstructive and idiopathic infertility [37,38]. This framework is not merely academic; it is the cornerstone of clinical decision-making, guiding the selection of diagnostic tests and tailoring therapeutic interventions, ranging from medical management to advanced assisted reproductive technologies. The first major category, obstructive infertility, accounts for approximately 40% of male infertility cases [39]. This condition is defined by a physical interruption—a blockage—anywhere along the male reproductive tract, which effectively prevents adequately produced sperm from being ejaculated. The testes typically demonstrate normal spermatogenesis, but the sperm are barred from their final destination. The urological causes of such obstructions are diverse. They can be congenital, such as CBAVD in cystic fibrosis, or, alternatively, obstructions can be acquired, most commonly resulting from iatrogenic causes like vasectomy or from post-infectious sequelae [40]. Infections such as epididymitis, gonorrhea, or chlamydia can lead to scarring and strictures within the delicate epididymis or vas deferens [41]. Trauma or surgical procedures in the pelvic region, such as hernia repair, can also inadvertently cause obstructive lesions [40]. In contrast, non-obstructive infertility, which constitutes 40–50% of cases, is characterized by a fundamental failure at the level of sperm production or function, despite a patent reproductive tract [42]. This category reflects testicular dysfunction and is notably heterogeneous in its etiology. A significant portion of non-obstructive cases stems from primary testicular failure, or hypergonadotropic hypogonadism, where the testes are unresponsive to pituitary gonadotropins [43,44]. This can be due to genetic disorders, such as the aforementioned Klinefelter syndrome or Y-chromosome microdeletions affecting AZF regions, or due to other prominent urological causes such as varicocele. Furthermore, testicular insult from trauma, torsion, or the toxic effects of chemotherapy and radiation are classic examples of non-obstructive infertility [45]. Despite a thorough evaluation, a significant proportion of cases, termed idiopathic male infertility, defy clear classification [46]. In these scenarios, standard semen analysis may reveal abnormalities like oligoasthenoteratozoospermia, but no specific obstructive or non-obstructive cause is identified. This diagnostic category underscores the limitations of our current understanding and diagnostic tools, hinting at subtle genetic, epigenetic, or environmental factors that impair sperm function—such as elevated DNA fragmentation or aberrant sperm epigenetics—without a macroscopic or readily identifiable lesion [47,48]. Given that a substantial majority of identifiable and treatable causes of male infertility fall squarely within the purview of urological practice—from surgically correctable varicoceles and obstructions to the hormonal management of hypogonadism—this manuscript will focus specifically on elucidating the diagnosis, management and contemporary treatment strategies for the urological causes of male infertility.

## 4. Key Urological Conditions Impacting Male Fertility

The management of male infertility is a cornerstone of modern urological practice. Many of the most common and treatable causes originate from disorders within the male genitourinary tract. These conditions can be conceptually divided into those that disrupt sperm production, those that obstruct sperm transport and those that interfere with the physical act of insemination [49,50]. It is important to note that endocrine disorders, such as male hypogonadism, are critical contributors to infertility. However, their evaluation and management often fall within a specialized medical endocrinology context. Therefore, they will not be the focus of this urologically oriented review [51,52,53]. Instead, this section will provide a comprehensive overview of the primary urological conditions—varicocele, obstructive azoospermia, erectile dysfunction and others—detailing their prevalence, pathophysiological mechanisms, diagnostic workup and contemporary treatment strategies (Table 2 and Table 3).

### 4.1. Varicocele

A varicocele, an abnormal dilation of the pampiniform venous plexus within the scrotum, is the most prevalent correctable cause of male infertility. It is found in approximately 15–20% of the general male population, but its significance is highlighted by its dramatically increased prevalence of 35–40% in men with primary infertility and up to 80% in those with secondary infertility [54,55]. The condition is most common on the left side due to anatomical factors, including the perpendicular insertion of the left testicular vein into the left renal vein, which creates higher venous pressure [56]. The pathophysiological impact on fertility is multifactorial, primarily mediated through testicular hyperthermia. The dysfunctional venous system impairs the counter-current heat exchange mechanism, leading to an elevated intratesticular temperature that is suboptimal for spermatogenesis. This is compounded by venous stasis and hypoxia, which promote oxidative stress and the accumulation of reactive oxygen species that damage sperm membranes and DNA integrity [57,58,59]. The clinical consequence is often reflected in semen analysis as a pattern of oligoasthenoteratozoospermia (reduced sperm count, motility and abnormal morphology) [60]. Diagnosis is primarily clinical via physical examination with the patient standing, often graded (I-III) based on palpability and visibility. Scrotal Doppler ultrasound is a valuable adjunct to confirm the diagnosis, assess venous diameter and document reflux [61]. Treatment is indicated in the context of infertility with a palpable varicocele and abnormal semen parameters. The goal is to ablate the dysfunctional veins, which can be achieved through microsurgical varicocelectomy—the gold standard due to its low complication and recurrence rates—or via percutaneous embolization by an interventional radiologist. Both methods are highly effective, with studies demonstrating significant improvements in semen parameters in 60–80% of men and consequent increases in spontaneous pregnancy rates [62,63,64].

### 4.2. Obstructive Azoospermia

Obstructive azoospermia is a condition where sperm production is normal, but a physical blockage prevents their appearance in the ejaculate. It accounts for approximately 40% of all azoospermia cases, which themselves affect about 1% of all men and 10–15% of infertile men [65]. The biological rationale is a straightforward mechanical interruption in the reproductive tract. Causes can be congenital, such as CBAVD, which is strongly associated with mutations in the CFTR gene (linked to cystic fibrosis), or acquired, such as from iatrogenic injury during hernia repair, post-infectious scarring from sexually transmitted infections or a prior vasectomy [66,67]. The diagnosis of obstructive azoospermia is suggested by a combination of normal testicular volume, palpable vasa deferentia (absent in CBAVD) and normal serum FSH levels, which indicate intact spermatogenesis. Semen analysis reveals azoospermia, sometimes with low ejaculate volume if the obstruction is at the level of the ejaculatory ducts. Transrectal ultrasound (TRUS) is crucial for evaluating the seminal vesicles and ruling out ejaculatory duct obstruction [68,69]. The treatment of obstructive azoospermia is one of the great success stories in reproductive urology. For suitable cases like post-vasectomy obstruction, microsurgical reconstruction via vasovasostomy or vasoepididymostomy offers high success rates for the return of sperm to the ejaculate [70,71]. When reconstruction is not feasible or fails, sperm retrieval techniques such as Microsurgical Epididymal Sperm Aspiration (MESA) or Testicular Sperm Extraction (TESE) can be performed [72,73]. The retrieved sperm are then used with Intracytoplasmic Sperm Injection (ICSI), enabling biological paternity for the vast majority of men with this condition [74].

### 4.3. Erectile Dysfunction

ED, the persistent inability to achieve and maintain an erection sufficient for satisfactory sexual performance, is a prevalent disorder whose incidence increases with age, affecting over 50% of men between 40 and 70 years old [75]. While ED does not directly impair spermatogenesis, it creates a direct mechanical barrier to natural conception by preventing successful intravaginal insemination [76,77]. Its etiology is multifactorial, encompassing vascular causes (e.g., diabetes, hypertension, cardiovascular disease), neurological disorders, hormonal imbalances (including hypogonadism, which itself affects sperm production) and psychological factors [78,79,80]. The diagnostic workup involves a detailed medical and sexual history, often aided by validated questionnaires like the International Index of Erectile Function (IIEF) [81]. A physical examination and targeted laboratory tests, including a hormonal profile, are essential to identify underlying conditions. Treatment is tailored to the etiology and the patient’s fertility goals. First-line therapy typically involves phosphodiesterase type 5 inhibitors (PDE5is) like sildenafil or tadalafil [82]. For men with hypogonadism, it is critical to avoid testosterone replacement therapy if fertility is desired, as it suppresses spermatogenesis; instead, treatments with gonadotropins or selective estrogen receptor modulators (SERMs) are preferred [83,84]. For cases refractory to medication, options include intracavernosal injections, vacuum erection devices, or, as a last resort, the surgical implantation of a penile prosthesis [85,86]. Addressing psychological components through counseling is also a vital aspect of a comprehensive treatment plan [87].

### 4.4. Peyronie’s Disease

Closely related to ED is PD, a condition characterized by the development of fibrous collagen plaques within the tunica albuginea of the penis. While its exact prevalence is uncertain, studies estimate it affects 3–9% of men, with incidence increasing with age [88]. The condition often presents in two phases: an acute inflammatory phase with penile pain and active plaque formation, followed by a chronic phase characterized by stable plaque and penile deformity [89,90,91]. The biological rationale for its impact on fertility is primarily mechanical. The resulting penile curvature, narrowing, or hinging can make sexual intercourse difficult, painful, or, in severe cases, impossible, thereby preventing effective sperm deposition [92]. Furthermore, the psychological distress associated with PD can contribute to secondary erectile dysfunction, compounding the problem [93]. In some cases, the fibrotic process can extend to involve the erectile tissue itself or the urethra, potentially affecting ejaculatory function [94]. Diagnosis is primarily clinical, based on patient history and physical examination, with penile ultrasound used to characterize plaque location and calcification [95,96]. Treatment is tailored to the disease phase and severity. In the acute phase, conservative treatments such as oral medications (e.g., Pentoxifylline) or intralesional injections (e.g., Collagenase Clostridium histolyticum) may be used [97]. For men with stable, debilitating deformities that preclude intercourse, surgical intervention is the mainstay. Options include penile plication (suturing to straighten the penis), plaque incision or excision with grafting, or, when ED is also present, penile prosthesis implantation [98]. By restoring the ability to have pain-free, penetrative intercourse, successful treatment of PD directly addresses the mechanical barrier to natural conception it creates [99].

### 4.5. Other Significant Urological Conditions

Beyond the major categories above, several other urological conditions significantly impact fertility. Cryptorchidism, or undescended testis, is a key congenital anomaly associated with impaired spermatogenesis and a higher risk of infertility and testicular malignancy, even after surgical correction—i.e., orchidopexy [100,101]. Testicular Torsion is a urological emergency most common in adolescents, where the spermatic cord twists, cutting off blood flow to the testis. The critical window for intervention is narrow, with the ideal time for surgical detorsion being within 6 h of symptoms onset to maximize testicular salvage. Even with prompt surgical detorsion, the ischemic and subsequent reperfusion injury can generate significant oxidative stress. This not only damages the affected testis but may also trigger an immunologic response that impairs spermatogenesis in the contralateral testis, leading to long-term infertility. Therefore, emergent surgical exploration and bilateral orchiopexy are standard. Given the significant risk to future fertility, long-term follow-up with semen analysis is strongly recommended for all post-puberal patients to assess and manage potential reproductive consequences [102,103]. Immunological infertility, characterized by the formation of antisperm antibodies (ASA), is another important mechanism [104]. ASA can arise secondary to conditions that breach the blood-testis barrier, such as testicular trauma, prior surgery, infections, tumors and the aforementioned torsion. These antibodies can impair sperm motility, cervical mucus penetration and fertilization [105,106]. Inflammatory conditions are another major category. Chronic Prostatitis, while not directly damaging spermatogenesis, contributes to an adverse seminal environment through leukocytospermia and oxidative stress, which can compromise sperm function [107,108]. Chronic Epididymitis and Orchitis, often resulting from bacterial infections (e.g., Chlamydia, Gonorrhea) or viral infections like mumps, can cause infertility through two primary mechanisms. The inflammatory process can lead to obstructive scarring within the delicate, convoluted tubules of the epididymis, blocking the passage of sperm. Concurrently, the inflammatory milieu itself, characterized by leukocytospermia and oxidative stress, can directly damage sperm membranes and DNA, reducing motility and fertilization potential [109,110,111,112,113]. Initial management prioritizes identifying the underlying cause, including appropriate screening for sexually transmitted infections, followed by targeted antibiotic or antiviral therapy alongside anti-inflammatory medications. In cases of persistent obstruction leading to azoospermia, surgical options such as epididymal reconstruction or, more commonly, sperm retrieval are available, with retrieved sperm used for ART [114,115,116]. Retrograde Ejaculation, where semen flows backwards into the bladder, is a challenging condition often caused by diabetes, spinal cord injuries or pelvic surgery [117]. Medical management with sympathomimetic agents is first-line, but sperm retrieval from post-ejaculation urine or directly from the bladder for use in ART may be necessary when pharmacotherapy fails [118,119]. Finally, Sperm DNA Fragmentation (SDF) is increasingly recognized as a key factor in male infertility, often underlying cases of unexplained infertility and recurrent pregnancy loss [120]. While not a “urological condition” in the traditional sense, it is a common pathological endpoint of several urological issues, most notably varicocele, as well as infections, exposure to environmental toxins and lifestyle factors like smoking [121]. Although assay-dependent, a DNA fragmentation index (DFI) exceeding 15–30% is generally considered abnormal and clinically significant [122]. Testing for SDF is particularly useful in specific clinical contexts, such as idiopathic infertility, recurrent pregnancy loss, or failed implantation after assisted reproductive technologies [123]. High levels of DNA fragmentation impair fertilization, embryo development and implantation [124]. Treatment focuses on addressing the root cause (e.g., varicocelectomy), implementing antioxidant therapy and utilizing advanced ART techniques like ICSI, which can help bypass this specific functional defect [122]. Given that oxidative stress from modifiable lifestyle factors is a primary driver of SDF, the assessment and management of SDF logically integrate with comprehensive lifestyle intervention programs, as detailed in Section 5.

## 5. The Role of Lifestyle Modifications in Male Fertility

Before discussing specific modifications, it is crucial to understand the central pathophysiological axis linking lifestyle to male infertility. This involves oxidative stress and its impact on sperm DNA. The highly mitotic and metabolically active process of spermatogenesis is exquisitely vulnerable to excess reactive oxygen species (ROS) [125,126]. Common lifestyle factors—poor diet, obesity, smoking, alcohol and psychological stress—directly or indirectly increase ROS production (oxidant load) and/or deplete endogenous antioxidant defenses [127,128]. The resulting state of oxidative stress damages spermatozoa at multiple levels: it peroxidizes lipid membranes (impairing motility and acrosome reaction), disrupts mitochondrial function (reducing energy production) and, most critically, induces breaks in the sperm DNA double helix [129,130]. SDF is a key functional defect that impairs embryonic development, reduces implantation rates and increases the risk of pregnancy loss, often manifesting clinically as idiopathic infertility or failed ART cycles [131,132]. Therefore, lifestyle interventions primarily aim to rebalance the seminal redox state by reducing oxidant triggers and boosting antioxidant capacity. Consequently, while the aforementioned urological, genetic and endocrine pathologies are primary diagnostic targets, a comprehensive approach to male infertility must also address these modifiable external factors that critically influence reproductive health. The management of male infertility has evolved to recognize that a patient’s daily habits and environment are not merely peripheral concerns but are central to his reproductive potential. A substantial body of evidence now firmly establishes modifiable lifestyle factors as critical determinants of semen quality and overall fertility status [133,134]. The European Association of Urology (EAU) guidelines explicitly recommend incorporating a detailed lifestyle assessment and targeted modification into the initial diagnostic and therapeutic algorithm for all men presenting with infertility, positioning it as a first-line, foundational intervention [135,136]. This paradigm shift is grounded in a clear pathophysiological rationale: the highly coordinated and hormonally sensitive process of spermatogenesis is uniquely vulnerable to oxidative stress, thermal insult and endocrine disruption [137,138]. Common lifestyle choices directly influence the production of ROS, sperm DNA integrity and the delicate balance of the hypothalamic-pituitary-gonadal (HPG) axis, thereby exerting a profound impact on semen parameters and the success rates of subsequent assisted reproductive technologies [139]. A comprehensive and multidisciplinary approach that empowers patients to adopt healthier lifestyles can significantly improve clinical outcomes and represents a cornerstone of modern andrological practice.

### 5.1. Dietary Patterns and Targeted Nutritional Supplementation

Dietary Patterns and Targeted Nutritional Supplementation form a critical pillar of fertility-focused lifestyle intervention. The overall dietary pattern appears to be more impactful than any single food. Adherence to a Mediterranean-style diet, characterized by abundant consumption of fruits, vegetables, whole grains, nuts, legumes, olive oil and fish, is consistently correlated with superior sperm motility, concentration and morphology. This is largely attributed to the diet’s high content of antioxidants and anti-inflammatory compounds, which counteract the oxidative stress known to damage sperm membranes and DNA [140,141,142,143]. Beyond general dietary patterns, evidence supports the use of targeted supplementation in specific clinical contexts. For men with idiopathic infertility, a trial of antioxidant supplements—such as vitamin C, vitamin E, zinc, selenium, coenzyme Q10 and carnitines—for a period of three to six months can lead to measurable improvements in semen parameters and potentially increase live birth rates [144,145,146]. However, clinical guidance is essential, as the indiscriminate use of over-the-counter supplements is not universally beneficial and should be informed by a patient’s specific nutritional status and clinical presentation, rather than adopted indiscriminately [147].

### 5.2. Weight Management and Physical Activity

The interconnected issues of Weight Management and Physical Activity are equally crucial in the optimization of male reproductive health. Obesity, particularly with a high body mass index (BMI) and increased visceral fat, is a well-established contributor to subfertility. The pathophysiological mechanisms are multifactorial, including the peripheral aromatization of testosterone into estrogen within adipose tissue, which suppresses the HPG axis and leads to hypogonadotropic hypogonadism [148]. Furthermore, obesity is associated with a state of chronic inflammation and increased scrotal temperatures, both of which are detrimental to spermatogenesis [149]. A structured weight loss program involving both dietary caloric restriction and increased physical activity has been demonstrated to improve testosterone levels and semen quality [150]. The role of physical activity itself is dose-dependent; moderate, regular exercise is associated with improved reproductive hormone profiles and reduced oxidative stress, thereby enhancing semen quality. Conversely, excessive endurance training, such as long-distance running or intensive cycling, can exert paradoxical detrimental effects due to elevated scrotal heat, hormonal imbalances and systemic inflammation [151,152]. Patients should therefore be advised to engage in consistent, moderate-intensity physical activity while consciously avoiding the pitfalls of overtraining.

### 5.3. Avoidance of Gonadotoxins

A non-negotiable component of any fertility optimization plan is the systematic avoidance of gonadotoxins. Cigarette smoking is one of the most well-established risk factors for male infertility, with strong evidence linking it to oligozoospermia, asthenozoospermia and significantly increased sperm DNA fragmentation. The toxicants in tobacco smoke directly damage testicular tissue and sperm cells, but the good news is that the effects are often reversible; smoking cessation has been shown to lead to measurable improvements in semen parameters within several months [153,154]. Similarly, excessive alcohol consumption and the use of recreational drugs—most notably cannabis, opioids and cocaine—have been demonstrated to suppress spermatogenesis and disrupt endocrine function [33,34,155,156]. Perhaps the most profound iatrogenic insult comes from the misuse of anabolic-androgenic steroids (AAS), which causes a profound and often prolonged suppression of the HPG axis, frequently resulting in azoospermia [35]. Cessation of AAS is imperative and recovery may be slow, sometimes requiring medical intervention with selective estrogen receptor modulators (SERMs) or gonadotropins to re-stimulate the testes [157]. Finally, men should be counseled on minimizing scrotal heat exposure from sources such as tight-fitting underwear, frequent hot tub or sauna use and prolonged sitting, as well as reducing occupational or environmental contact with endocrine-disrupting chemicals like bisphenol A (BPA), phthalates and pesticides, which have been implicated in the global decline of semen quality [158,159,160].

### 5.4. Management of Stress and Sleep Hygiene

**Figure 1:**
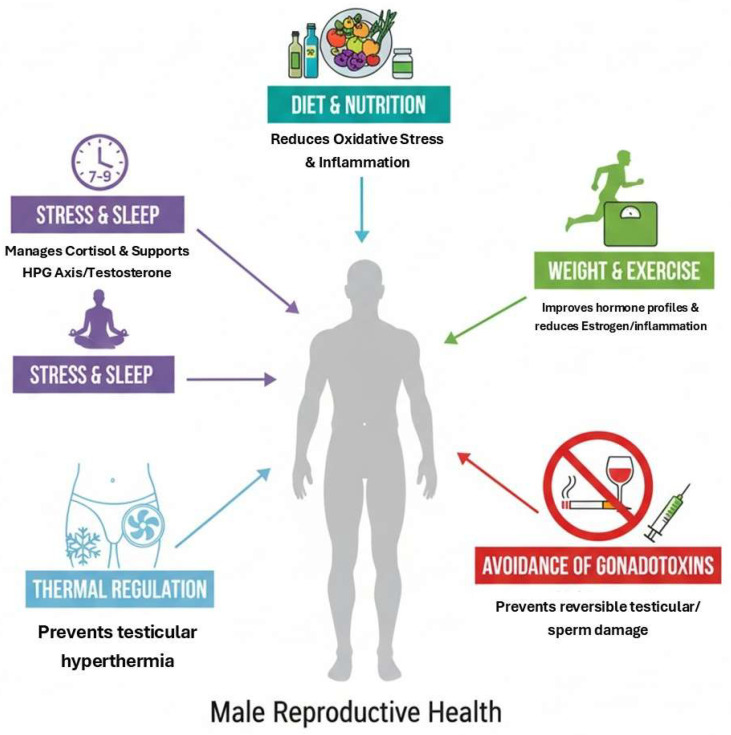
Evidence-Based Lifestyle Modifications to Improve Male Reproductive Health.

Managing psychological stress and sleep hygiene is an often underappreciated yet vital aspect of a holistic fertility plan. Chronic psychological stress disrupts the delicate interplay between the Hypothalamic–Pituitary–Adrenal axis (HPA) (cortisol production) and the hypothalamic–pituitary–gonadal (HPG) axis (testosterone production), leading to hormonal imbalances that can negatively impact spermatogenesis. Furthermore, sleep disturbances and circadian misalignment are independently associated with reduced testosterone levels and poorer semen quality. Simple, actionable advice—such as incorporating mindfulness-based stress reduction techniques, ensuring a consistent sleep schedule and prioritizing 7–9 h of quality sleep per night—can serve as powerful, low-risk adjuncts to a comprehensive fertility strategy (Figure 1) [161,162].

### 5.5. A Stepwise Clinical Protocol for Lifestyle Optimization

Translating the evidence linking lifestyle, oxidative stress and SDF into clinical practice requires a shift from general advice to a structured, actionable protocol. The overarching goal is to systematically reduce the patient’s seminal oxidant load while enhancing endogenous antioxidant defenses [163]. A recommended approach, designed for seamless integration into the initial fertility workup, consists of three consecutive phases: comprehensive assessment, phenotype-specific intervention and active monitoring.

The initial phase involves a comprehensive baseline assessment during the first consultation. This extends beyond standard andrological metrics—such as BMI, waist circumference and blood pressure—to include a targeted lifestyle inventory. A brief questionnaire can efficiently capture key modifiable domains: diet quality, smoking status, alcohol and recreational drug use (with specific attention to anabolic steroids), exercise habits, occupational exposures to heat or chemicals, sleep hygiene and perceived stress levels. In specific clinical contexts—particularly idiopathic infertility, recurrent pregnancy loss, or prior failure of assisted reproductive technologies—the diagnostic workup should strongly consider incorporating an assessment of SDF, permitting the quantification of the functional impact of oxidative stress, thus providing a critical baseline against which to measure the efficacy of subsequent interventions [164].

The second phase entails phenotype-specific, targeted counseling, where interventions are personalized based on the assessment profile. For patients presenting with obesity, a high BMI or signs of systemic inflammation, the counseling focus should be a structured weight loss program targeting a 5–10% reduction in body weight, emphasizing a calorie-restricted anti-inflammatory diet such as the Mediterranean pattern, combined with regular moderate-intensity aerobic exercise [142,150]. This strategy directly mitigates adipose-tissue-mediated aromatization and chronic inflammation. For patients with elevated SDF or idiopathic infertility, counseling must aggressively target oxidative stress through strict gonadotoxin cessation (prioritizing smoking cessation and elimination of anabolic steroid use), a prescribed 3-to-6-month course of evidence-based antioxidant combinations (e.g., vitamin C, vitamin E, zinc, selenium, coenzyme Q10, or carnitines) and practical advice for scrotal cooling, such as avoiding tight-fitting underwear and prolonged sitting [144,165]. For individuals with significant psychological stress or sleep disturbances, recommendations should include evidence-based stress-reduction techniques, including mindfulness-based programs and structured sleep hygiene interventions to help stabilize the hypothalamic–pituitary–adrenal axis [166,167].

The final, crucial phase is active monitoring and reinforcement. Lifestyle modification is a behavioral process that requires ongoing support. Scheduling a dedicated follow-up visit 3 to 6 months after the initial intervention serves to reinforce positive behavior changes, address practical barriers and objectively reassess progress through repeat semen analysis and, if initially elevated, SDF testing. This follow-up data is invaluable for sustaining patient motivation and for making informed decisions regarding the next steps in the clinical pathway, such as proceeding with surgical correction of a varicocele or initiating assisted reproductive technologies. By adopting this structured protocol, lifestyle intervention is elevated from a peripheral suggestion to a foundational, targeted and monitorable pillar of male infertility management. It enables a personalized therapeutic approach that directly addresses the modifiable oxidative stressors impairing sperm function and genomic integrity.

## 6. The Role of Assisted Reproductive Technologies

When conventional medical or surgical interventions for male infertility are unsuccessful, contraindicated, or unlikely to succeed, ART provides a powerful pathway to biological parenthood. These advanced techniques represent the culmination of the infertility treatment journey, offering solutions even for the most severe male factor diagnoses [168]. The selection of a specific ART modality is tailored to the underlying cause and severity of the male infertility. Intrauterine Insemination (IUI), the least complex technique, involves the laboratory processing of a semen sample to concentrate motile sperm, which are then placed directly into the uterus around the time of ovulation [169]. IUI is typically reserved for mild male factor cases, such as mild oligospermia or asthenospermia, when the female partner’s fertility is uncompromised. For more significant male factor infertility, In Vitro Fertilization (IVF) is employed. This process involves retrieving eggs from the female partner and fertilizing them with sperm in a laboratory dish. The resulting embryos are then transferred to the uterus [170]. The most transformative advancement in this field is ICSI, a specialized form of IVF. ICSI involves the direct injection of a single sperm into a mature egg, effectively bypassing all natural barriers to fertilization. This technique has revolutionized the treatment of severe male infertility, including conditions like obstructive and non-obstructive azoospermia, extremely low sperm counts, or poor sperm motility [171]. The efficacy of ICSI is such that it can be successfully performed with sperm retrieved directly from the testes (TESE) or epididymis (MESA), making biological fatherhood possible for men who were once considered irreversibly infertile [172,173]. While ART and particularly ICSI, offer remarkable hope, it is generally considered after other urological and lifestyle interventions have been explored, due to its more invasive nature, high cost and the need for concomitant female partner treatment. Ultimately, the integration of ART into the treatment arsenal ensures that a vast majority of couples facing male infertility have a viable route to achieving their reproductive goals [173,174].

## 7. Conclusions

Male infertility is a multifactorial condition where urological causes play a predominant and often treatable role. This review has delineated the significant impact of conditions such as varicocele, obstructive azoospermia, erectile dysfunction, and Peyronie’s disease on male reproductive potential, underscoring the necessity of a systematic and thorough urological evaluation. A modern diagnostic approach, integrating medical history, physical examination, semen analysis, and targeted diagnostics, is crucial for identifying the underlying etiology. The cornerstone of contemporary management is a logical, stepwise progression designed to optimize outcomes: it begins with foundational lifestyle modifications, advances to targeted medical or surgical interventions, and culminates, when necessary, in the application of assisted reproductive technologies. This tiered strategy ensures that care is both efficient and comprehensive, leveraging all available tools from basic wellness to advanced ART. Ultimately, a holistic and multidisciplinary approach—bridging urology, endocrinology, reproductive medicine, and nutritional and psychological support—is paramount. Such an integrated model ensures that men receive personalized, comprehensive care addressing both biological and psychological well-being on their path to parenthood.

## Figures and Tables

**Table 1:** Genetic and Environmental Risk Factors.

Category	Examples	Mechanism of Impact on Fertility
**Genetic Factors**		
Chromosomal	Klinefelter Syndrome (47, XXY)	Causes testicular fibrosis and primary testicular failure (azoospermia).
Monogenic	CFTR mutations (CBAVD), Y-chromosome microdeletions (AZF regions)	Causes obstructive azoospermia or disrupts specific stages of spermatogenesis.
Epigenetic	Altered DNA methylation/histone modification	Paternal lifestyle can cause transmissible changes affecting embryo/offspring health.
**Environmental & Lifestyle Factors**		
Endocrine Disruptors	Pesticides, Phthalates	Mimic or block hormones, disrupting the hypothalamic–pituitary–gonadal axis
Physical Hazards	Heat, Ionizing Radiation	Directly damages spermatogonia, impairing sperm production
Lifestyle	Obesity, Smoking, Excessive Alcohol, Anabolic Steroids	Causes oxidative stress, hormonal suppression, inflammation and direct sperm damage.
Paternal Age	Advanced Age	Gradual decline in semen quality, increased DNA fragmentation and de novo mutations

**Table 2:** Key Urological Conditions: Pathophysiology and Diagnosis.

Condition	Prevalence	Pathophysiology	Diagnostic Workup
Varicocele	15–20% general population; 35–40% primary infertility; 80% secondary infertility	Testicular hyperthermia, venous stasis, hypoxia and oxidative stress damaging sperm	Physical exam, scrotal Doppler ultrasound
Obstructive Azoospermia	40% of azoospermia cases	Physical blockage preventing sperm passage despite normal production.	Azoospermia on semen analysis, normal testicular volume, normal FSH
Erectile Dysfunction	>50% of men aged 40–70	Mechanical barrier to conception. Multifactorial etiology (vascular, neurological, hormonal, psychological)	Medical history, sexual history, IIEF questionnaire, physical exam, hormonal profile
Peyronie’s Disease	3–9% of men	Fibrous plaques cause penile curvature, making intercourse difficult or impossible.	Medical history, physical exam, penile ultrasound
Cryptorchidism	~1% of males at 1 year; higher in premature infants	Abnormal testicular descent leads to impaired germ cell development, hyperthermia and increased risk of malignancy, even after correction.	Physical exam, scrotal/abdominal ultrasound. Diagnosis is clinical.
Testicular torsion	Urological emergency	Ischemia–reperfusion injury causes oxidative stress & potential immunologic damage to both testes.	Clinical diagnosis, scrotal Doppler ultrasound, surgery
Immunological Infertility	Variable; associated with barrier disruption	Breach of the blood-testis barrier leads to antisperm antibody formation, impairing sperm motility and function.	Semen analysis with mixed antiglobulin reaction test or immunobead test for antisperm antibody detection.
Chronic Prostatitis	Common in adult males	Chronic inflammation contributes to a hostile seminal environment via leukocytospermia and elevated oxidative stress, compromising sperm function.	Medical history, physical exam, semen analysis and cultures.
Chronic Epididymitis/Orchitis	Post-infectious	Obstructive scarring and inflammatory damage to sperm	Medical history, physical exam, sperm cultures, scrotal Doppler ultrasound
Retrograde Ejaculation	Relatively rare but significant cause	Failure of bladder neck closure during emission, leading to semen flowing into the bladder. Can be caused by diabetes, surgery, neurological disorders or medications.	Post-ejaculatory urinalysis (presence of sperm). Medical history for identifying etiology.
Sperm DNA Fragmentation	Common endpoint of many insults	Damaged sperm DNA impairs embryo development and implantation.	Specialized sperm function tests

**Table 3:** Key Urological Conditions: Management Strategies.

Condition	Treatment Options	Key Considerations
Varicocele	Microsurgical Varicocele or Percutaneous Embolization	Improve semen parameters in 60–80% of men
Obstructive Azoospermia	Microsurgical reconstruction or Sperm Retrieval	Reconstruction is the first choice if feasible. Sperm retrieval + ICSI enables paternity for most men
Erectile Dysfunction	PDE5 inhibitors as first line; Gonadotropins/SERMs for hypogonadism; Intracavernosal injections, vacuum devices and penile prosthesis as second and third line	Tailor to etiology and fertility goals
Peyronie’s Disease	Oral meds and intralesional injections during the acute phase, while surgery for chronic phase	Restore ability to have penetrative intercourse
Cryptorchidism	Surgical Orchidopexy, typically performed between 6 and 18 months of age.	Early surgical correction is crucial to preserve fertility potential and facilitate cancer surveillance, though some impairment of spermatogenesis may persist
Testicular torsion	Emergent surgical detorsion & bilateral orchiopexy	Salvage the testis and protect contralateral testis from immunologic damage. Time is critical: surgery ideally within 6 h of symptom onset
Immunological Infertility	Corticosteroids. Utilize ART	Medical immunosuppression has limited efficacy and significant risks. ART is the most effective strategy to overcome antibody-mediated impairment of fertilization
Chronic Prostatitis	Antibiotics, alpha-blockers, anti-inflammatory agents and supportive measures	Focus is on symptom control and reducing the pro-inflammatory seminal environment. A direct causal link to infertility is debated, but treatment may improve sperm quality
Chronic Epididymitis/Orchitis	Antibiotics/Antivirals for infectious cases; otherwise, anti-inflammatories and analgesics. Surgery or ART for persistent obstruction	Management depends on etiology. A significant challenge is diagnosing and managing nonbacterial/idiopathic cases. The primary goals are to resolve active infection/inflammation and to bypass any resulting obstruction.
Retrograde Ejaculation	Medical Therapy. Sperm Retrieval from post-ejaculation urine or via bladder catheterization for use in ART	Pharmacotherapy aims to restore antegrade ejaculation. When medication fails, retrieval of viable sperm from urine for ART
Sperm DNA fragmentation	Treat root cause, antioxidants, ART	Reduce DNA damage and use ART to bypass the functional defect.

## Data Availability

No new data were created or analyzed in this study.
